# A disease warranting attention from neurosurgeons: primary central nervous system post-transplant lymphoproliferative disorder

**DOI:** 10.3389/fneur.2024.1392691

**Published:** 2024-05-15

**Authors:** Lei Jin, Di Lu, Feng Yan, Jinkun Han, Penghu Wei, Yiqiang Zhou, Yaming Wang, Yongzhi Shan, Guoguang Zhao

**Affiliations:** ^1^Department of Neurosurgery, Xuanwu Hospital, Capital Medical University, Beijing, China; ^2^Clinical Research Center for Epilepsy, Capital Medical University, Beijing, China; ^3^Beijing Municipal Geriatric Medical Research Center, Beijing, China

**Keywords:** primary central nervous system post-transplant lymphoproliferative disorder, kidney transplant, hematopoietic stem cell transplantation, robot-assisted stereotactic brain biopsy, brain tumor

## Abstract

**Background:**

Primary central nervous system post-transplant lymphoproliferative disorder (PCNS-PTLD) is a rare condition, posing diagnostic and treatment challenges, with histological biopsy essential for diagnosis. Standardized treatment protocols are lacking. This disease requires urgent attention due to the increasing number of organ transplant surgeries and the use of immunosuppressive agents.

**Methods:**

From 2020 to 2023, our center diagnosed five patients with PCNS-PTLD. We reviewed their clinical records and conducted a comprehensive analysis of 22 literatures on PCNS-PTLD cases following renal transplantation or allogeneic hematopoietic stem cell transplantation (HSCT).

**Results:**

Four patients had previously received a kidney transplant, one had undergone allogeneic HSCT. The median time from the last transplant surgery to the diagnosis of PCNS-PTLD differs between kidney transplant (21.5 years) and allogeneic HSCT (9 months). Common symptoms included motor weakness (*n* = 4), headache (*n* = 2), confusion (*n* = 2), and nausea (*n* = 2), with ring-enhancing (*n* = 5), typically solitary (*n* = 3) and supratentorial (*n* = 3) lesions on imaging. Diagnosis involved robot-assisted stereotactic brain biopsy (*n* = 4) or craniotomy (*n* = 1), all showing Epstein-Barr virus and CD20 positivity. Most cases (*n* = 4) were monomorphic diffuse large B-cell lymphoma. Treatment included rituximab (*n* = 3), surgical resection (*n* = 2), zanubrutinib (*n* = 1), whole-brain radiation (*n* = 1), and methotrexate (*n* = 1). At the last follow-up, the median duration of follow-up for all patients was 19 months. During this time, 3 patients had died and 2 patients were still alive.

**Conclusion:**

In patients with a history of kidney transplantation or allogeneic HSCT who are on long-term immunosuppressive therapy, any neurological symptoms, particularly the presence of supratentorial ring-enhancing masses in the brain on imaging, whether solitary or multiple, should raise high suspicion for this disease, warranting a timely brain biopsy. Additionally, we found that besides reducing immunosuppressants, zanubrutinib may be a potential, safe, and effective treatment for this condition. Moreover, post-surgical administration of rituximab in conjunction with whole-brain radiotherapy also appears to be a potentially safe and effective approach.

## 1 Introduction

Post-transplant lymphoproliferative disorder (PTLD) refers to a group of lymphoproliferative disorders that occur in recipients of solid organ transplantation (SOT) or hematopoietic stem cell transplantation (HSCT) under pharmacologic immunosuppression (1). Although about 7%–15% of all PTLD patients involve the central nervous system (CNS), primary involvement of the nervous system is quite rare (2, 3). Primary CNS post-transplant lymphoproliferative disorder (PCNS-PTLD) is defined as a lymphoproliferative disorder limited to the CNS occurring after SOT or HSCT, with no evidence of systemic PTLD (2, 4). The incidence rate of PCNS-PTLD is approximately 11.5 per 100,000 person-years (3). Due to its extreme rarity, diagnosing and treating PCNS-PTLD pose significant challenges (5). Currently, there are no standard treatment protocols for PCNS-PTLD, and management relies solely on existing case reports and small retrospective case series to guide therapy (6). Additionally, to our knowledge, there have been no independent case series reports of PCNS-PTLD following HSCT to date.

With an increasing number of successful organ transplant surgeries, the growing use of immunosuppressive agents, and the introduction of new immunosuppressive drugs, PCNS-PTLD is expected to occur more frequently (4, 7, 8). Consequently, it is imperative for medical professionals, especially neurosurgeons, to be aware of this condition and maintain a high index of suspicion to initiate appropriate treatment before disease progression (9). Here, we present a single-institution case series of 5 patients, 4 of whom developed PCNS-PTLD after kidney transplantation, and 1 after allogeneic HSCT. Additionally, we reviewed 22 case reports and case series of PCNS-PTLD following kidney transplantation or allogeneic HSCT, focusing on clinical presentations, radiological features, pathological characteristics, treatment modalities, and prognosis to aid in the better diagnosis and management of this disease in the future.

## 2 Patients and methods

We conducted a thoroughly retrospective review of all clinical records from the Department of Neurosurgery at Xuanwu Hospital, Capital Medical University, from January 2020 to December 2023, and identified a total of 5 patients diagnosed with PCNS-PTLD after kidney or allogeneic HSCT over the past 3 years. Among them, four patients were diagnosed via robot-assisted stereotactic brain biopsy, while pathology findings from tumor resection during craniotomy confirmed the diagnosis in one patient. All patients underwent staging computed tomography (CT) imaging or whole-body postoperative positron emission tomography-computed tomography (PET-CT) to rule out systemic involvement of lymphoma. This study primarily reviewed preoperative clinical symptoms, preoperative magnetic resonance imaging (MRI), or CT scan for each patient. We also examined the immunosuppressive agents used by each patient at the onset of clinical symptoms, treatments administered after diagnosis, and their survival status. Basic information for each patient, including age at onset of clinical symptoms and time interval from last transplantation to onset of clinical symptoms, was collected and is presented in Table 1. The last follow-up date was January 9, 2024.

**Table 1:** Main characteristics of this case series.

**No**.	**Age at diagnosis, median**	**Sex**	**Time from the most recent transpl- antation to diagnosis, median, y**	**Type of transpl-antation**	**Neurolo- gical symptoms**	**Immuno- suppre- ssive agents^#^**	**Lesions**	**Exact lesion location**	**Lesion location**	**Lesion enhanc-ement**	**Histologi- cal subtype**	**Treatments**	Overall survival*	**Survival status**
1	57	Male	6 y	Kidney	Left motor weakness	Tacrolimus, mycophenolate, prednisone	Solitary	Right frontal lobe	Supra.	Ring enhancement	Polymorphic PTLD	ROI, rituximab, resection	3 y	Die (non-PTLD)
2	13	Female	9 m	Allogeneic HSCT	Left motor weakness, headache, facial droop	Tacrolimus, mycophenolate, prednisone	Multiple	Right basal ganglia, left cerebellum	Supra. & infra.	Ring enhancement	Monomorphic, DLBCL	ROI, rituximab, WBRT, resection	42 m	Alive
3	59	Female	24 y	Kidney	Right motor weakness, confusion, speech difficulties, depression mood	Tacrolimus, mycophenolate, prednisone	Solitary	Left midbrain cerebral peduncle, thalamus, basal ganglia	Supra. & infra.	Ring enhancement	Monomorphic, DLBCL	Rituximab	3 m	Die (non-PTLD)
4	62	Female	19 y	Kidney	Headache, nausea	Cyclosporine, mycophenolate, prednisone	Solitary	Right basal ganglia	Supra.	Ring enhancement	Monomorphic, DLBCL	ROI, zanubrutinib	19 m	Alive
5	52	Female	27 y	Kidney	Confusion, right motor weakness, nausea, right visual field defect	Cyclosporine, mycophenolate, prednisone	Multiple	Bilateral parietal lobe	Supra.	Ring enhancement	Monomorphic, DLBCL	ROI, MTX	18 d	Die (PTLD)

d, days; DLBCL, diffuse large B-cell lymphoma; HSCT, hematopoietic stem cell transplantation; infra, infratentorial; m, months; MTX, methotrexate; NR, not report; PTLD, post-transplant lymphoproliferative disorder; ROI, reduction of immunosuppression; supra, supratentorial; WBRT, whole—brain radiation therapy; y, years.

^*^Represents the time from date of clinical symptoms appear to date of last follow-up or die.

^#^Represents the immunosuppressive agents at the time of PTLD diagnosis.

Furthermore, we conducted a literature review on PCNS-PTLD occurring after kidney or HSCT. Two independent researchers screened titles and abstracts of published manuscripts available on PubMed (https://pubmed.ncbi.nlm.nih.gov/) from the establishment of the database until December 2023. Inclusion criteria required manuscripts to be published in English, meet the definition of PCNS-PTLD (2, 4), involve patients who underwent kidney or HSCT before the onset of clinical symptoms, and qualify as case reports or case series. The search strategy involved combinations of the following terms: “primary central nervous system post-transplant lymphoproliferative disorder”, “PCNS-PTLD”, “kidney transplant”, “HSCT”, “renal transplant”, and “hematopoietic stem cell transplantation”. Ultimately, 22 manuscripts were selected, reporting on a total of 45 patients. Based on the descriptions provided in the literature, we summarized and compiled Table 2.

**Table 2:** Main characteristics of 22 reviewed literatures.

**References**	***N***	**Age at diagnosis, median**	**Time from the most recent trans- plantation to diagnosis, median**	**Type of trans- plantation**	**Neurolo- gical symptoms**	**Immuno- suppressive agents^#^**	**Lesions**	**Lesion location (suprate- ntorial or infra- tentorial)**	**Imaging charact- eristics**	**Histo- logical subtype**	**Treatments**	Overall survival^*^, median	**Survival status**
1. Lake et al. (4)	10	49	4.5 y	7 kidney 3 kidney/pancreas	Confusion, headache, speech difficulties, facial droop, diplopia, motor weakness, vertigo, ataxia, seizure	Cyclosporine, tacrolimus, mycophenolate, prednisone	3 solitary 7 multiple	8 Supra. 1 infra. 1 supra. & infra.	Ring enhancement, incomplete ring	4 polymorphic, 1 monomorphic, peripheral T cell 5 monomorphic, DLBCL	ROI, dexamethasone, rituximab, MTX, TMZ, WBRT	2.75 y die 1.75 y alive	4 die 6 alive
2. Sola-Valls et al. (7)	5	44	84 m	3 kidney 2 kidney/pancreas	Headache, seizure, gait disturbance, confusion, hallucinations, dysarthria	Mycophenolate, prednison, tacrolimus, cyclosporine	5 multiple	3 supra. 2 supra. & infra.	Ring enhancement	monomorphic※	ROI, rituximab, WBRT, MTX, TMZ	9 w die 26 m alive	4 die 1 alive
3. Ishihara et al. (10)	6	60	88.3 m	Kidney	NR	Cyclosporine, azathioprine, methylprednisolone, tacrolimus, mycophenolate	NR	NR	NR	1 polymorphic 1 monomorphic, malignant lymphoma 4 monomorphic, DLBCL	ROI, MTX, WBRT, cytarabine-etoposide, R-CHOP, resection	39.25 m alive 3.9 m die	4 alive 2 die
4. Huang et al. (9)	5	35.3	12 y	Kidney	Headache, sleepy, depression mood, consciousness, disturbance, nausea, vomiting, coma, tremor, seizure, left limbs weakness, palsy	Azathioprine, cyclosporine, tacrolimus, mycophen, prednisolone	NR	NR	Ring enhancement	5 monomorphic, DLBCL	ROI, WBRT, resection	36.5 m (alive) ^**^NR (die)	4 alive 1 die
5. Law et al. (11)	2	36	4 y	1 kidney 1 kidney/pancreas	Seizure, visual field defect	NR	1 solitary 1 multiple	1 supra. 1 NR	NR	2 monomorphic, DLBCL	ROI, rituximab, MTX, ibrutinib, EBV-specific T cells	25.5 m	Alive
6. Yeung et al. (12)	1	41	26 y	Kidney	Seizure, headache	Cyclosporine, mycophenolate, prednisone	Multiple	Supra. & infra.	NR	Monomorphic, DLBCL	ROI, rituximab, MTX, WBRT	12 m	Alive
7. Xu et al. (2)	1	68	5 y	Kidney	NR	Mycophenolate, tacrolimus	Multiple	Supra.	Enhancing	Monomorphic, DLBCL	ROI, rituximab	NR	Alive
8. Brennan et al. (13)	1	6	3 y	Kidney	Headache, nausea, vomiting, seizure	NR	Multiple	Supra.	Ring enhancement	Monomorphic, DLBCL	ROI, acyclovir	26 m	Alive
9. Valencia-Sanchez et al. (8)	1	55	NR	Kidney	Gait instability, vertigo, dysarthria	Mycophenolate, prednison	Multiple	Supra. & infra.	Ring enhancement	Monomorphic, DLBCL	ROI, rituximab, cyclophps- phamide, doxorubicin, vincristine, prednisone, WBRT	17 m	Die
10. Teresa et al. (14)	1	NR	11 y	Kidney	Weakness, memory loss, expressive dysphasia	Mycophenolate, prednison	Solitary	Supra.	Ring enhancement CT	Monomorphic, DLBCL	ROI, WBRT, rituximab	30 m	Alive
11. Imafuku et al. (15)	1	62	4 y	Kidney	Peripheral palsy, bilateral sensorineural hearing loss	Tacrolimus, mycophenolate, prednisone	Multiple	Supra. & infra.	Ring enhancement	Monomorphic, DLBCL	ROI, MTX, cytarabine, WBRT, rituximab	18 m	Alive
12. Reis et al. (16)	1	37	10 y	Kidney	Confusion, hemiparesis	Mycophenolate, prednisone, tacrolimus	Solitary	Supra.	Ring enhancement	Monomorphic※	ROI	NR	Alive
13. Tanaka et al. (17)	1	62	13 y	Kidney	Speech disturbance	Prednisolone, tacrolimus, mycophenolate	Solitary	Supra.	Ring enhancement	Monomorphic, DLBCL	ROI, rituximab	34 m	Alive
14. Azriel et al. (18)	1	49	18 y	Kidney	Headache, nausea, declining visual acuity	Prednisolone, cyclosporine, mycophenolate	Solitary	Supra.	Ring enhancement	Hodgkin lymphoma PTLD	Rituximab, MTX, procarbazine, vincristine, resection	NR	NR
15. Said-Conti et al. (19)	1	11	82 m	Kidney	Seizure, depressed neurological state	Prednisolone, tacrolimus, mycophenolate	Multiple	Supra.	Ring enhancement	Polymorphic	ROI, hydroxyurea, rituximab, WBRT	3 y	Alive
16. Yaginuma et al. (20)	1	36	5 y	Kidney	Consciousness alter	Tacrolimus, mycophenolate, prednisone	Multiple	Supra.	Ring enhancement	Monomorphic, DLBCL	ROI, WBRT	29 m	Alive
17. Kittan et al. (21)	1	49	6 m	Allogeneic HSCT	Confusion, diplopia, ataxia	Prednisolone, mycophenolate	Multiple	Supra. & infra.	Ring enhancement	Monomorphic, B cell lymphoma※	Rituximab, foscarnet, DLI	8 w	Die
18. Aisa et al. (22)	1	58	340 d	Allogeneic HSCT	Headache, consciousness loss	Cyclosporin, MTX, prednisolone, mycophenolate	Multiple	Supra.	-	Monomorphic, DLBCL	-	8 d	Die
19. Toyosaki et al. (23)	1	32	4 m	Allogeneic HSCT	Convulsion	Tacrolimus, prednisolone, cyclophosphamide	Multiple	Supra.	Ring enhancement	Monomorphic, DLBCL	ROI, high does MTX	NR	Die
20. Kassa et al. (5)	1	11	82 d	Allogeneic HSCT	Nausea, vomiting, diplopy	Mycophenolate	Multiple	Supra. & infra.	-	Monomorphic, DLBCL	ROI, nivolumab	1 y	Alive
21. Mayumi et al. (24)	1	51	306 d	Allogeneic HSCT	Hemiplegia	Prednisolone, tacrolimus	Solitary	Supra.	Ring enhancement	Monomorphic, DLBCL	ROI, rituximab, DLI	355 d	Alive
22. Sakamoto et al. (25)	1	17	621 d	Allogeneic HSCT	Seizure	Tacrolimus, prednisolone, mycophenolate	Solitary	Supra.	Ring enhancement	NR	ROI, rituximab, WBRT	397d	Die

d, days; DLBCL, diffuse large B-cell lymphoma; DLI, donor lymphocyte infusion; HSCT, hematopoietic stem cell transplantation; infra, infratentorial; m, months; MTX, methotrexate; NR, not report; PTLD, post-transplant lymphoproliferative disorder; R-CHOP, rituximab, cyclophosphamide, hydroxydaunorubicin, oncovin, prednisone; ROI, reduction of immunosuppression; supra, supratentorial; TMZ, temozolomide; WBRT, whole—brain radiation therapy; y, years.

※ Represents the specific type of lymphoma was not specified in the literature, only mentioned as monoclonal.

^*^Represents the time from date of clinical symptoms appear to date of last follow-up or die.

^**^Represents the average survival time.

^#^Represents the immunosuppressive agents at the time of PTLD diagnosis.

## 3 Illustrative cases

### 3.1 Case 1

**Figure 1:**
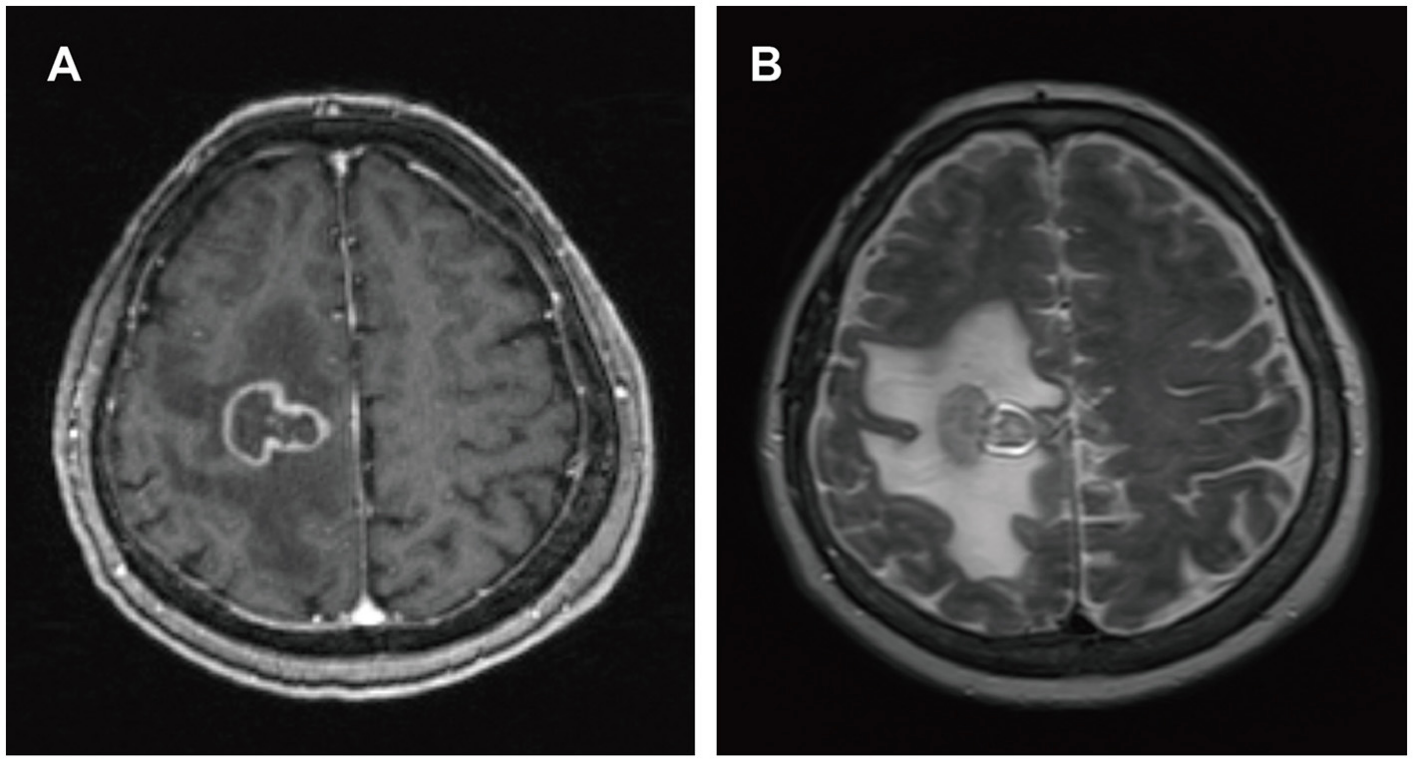
MRI findings of patient 1. Irregular ring enhancement lesion in the right frontal lobe on T1-weighted images **(A)**. Extensive perilesional edema in the right frontal lobe on T2-weighted images **(B)**.

**Figure 2:**
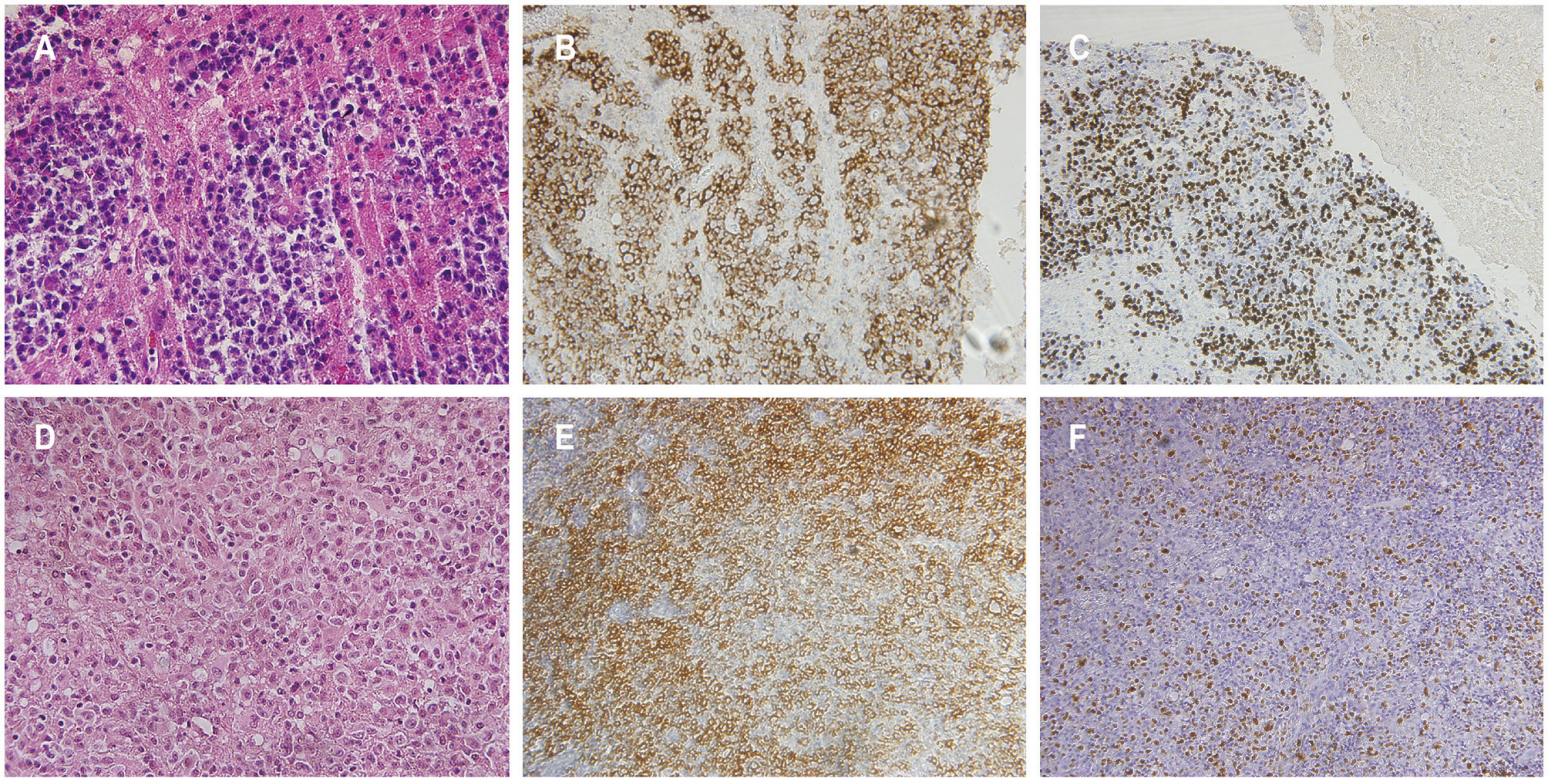
Histopathological features of patients 1 and 3. Hematoxylin and eosin staining in patient 1 [**(A)** magnification × 400]. The atypical cells demonstrated positive staining for CD20 in patient 1 **(B)**. Detection of Epstein-Barr virus (EBV) encoded small RNA positive cells was observed in patient 1 **(C)**. Hematoxylin and eosin staining in patient 3 exhibited sheets of atypical cells with prominent nuclear division, visible nucleoli in some cells, and extensive areas of necrosis, indicative of monomorphic PTLD, diffuse large B-cell lymphoma [**(D)** magnification × 400]. The atypical cells in patient 3 stained positive for CD20 **(E)**. Presence of cells positive for EBV-encoded small RNA in patient 3 was confirmed **(F)**.

A 57-year-old male presented to a local hospital with a two-month history of progressive weakness in the left limbs, characterized by leftward deviation while walking. He underwent a kidney transplant six years prior due to chronic renal failure and has been on a regimen of tacrolimus, mycophenolate mofetil, and prednisone as immunosuppressive therapy since then. There is no personal or family history of malignancies. He denied headaches, dizziness, weight loss, fever, or any prodromal symptoms. A head CT scan revealed a heterogeneous density nodular lesion in the right frontal lobe with finger-like brain edema. Contrast-enhanced MRI of the head exhibited ring-enhancing lesions in the right frontal lobe with surrounding extensive edema (Figure 1). Over the past ten days, weakness in his left upper limb progressively worsened, resulting in the inability to move his left hand, although he could elevate his left upper limb but couldn't raise it above the shoulder. Consequently, he underwent right frontal lobe lesion resection at our neurosurgery department. Postoperatively, there was improvement in the weakness of the left limbs compared to preoperative status. Histopathological and immunohistochemical studies of the lesion confirmed the diagnosis of PTLD, with a leaning toward polymorphic PTLD (Figure 2). Tumor cells stained positive for CD20 and CD79a. Epstein-Barr virus-encoded RNA (EBER) was positive. CT scans of the neck, chest, abdomen, and pelvis showed no abnormalities. The patient was ultimately diagnosed with PCNS-PTLD. He underwent reduction of immunosuppression combined with rituximab therapy. Follow-up MRI of the head postoperatively revealed no recurrence of the disease. Approximately three years after being diagnosed with PCNS-PTLD, he passed away due to myocardial infarction.

### 3.2 Case 2

**Figure 3:**
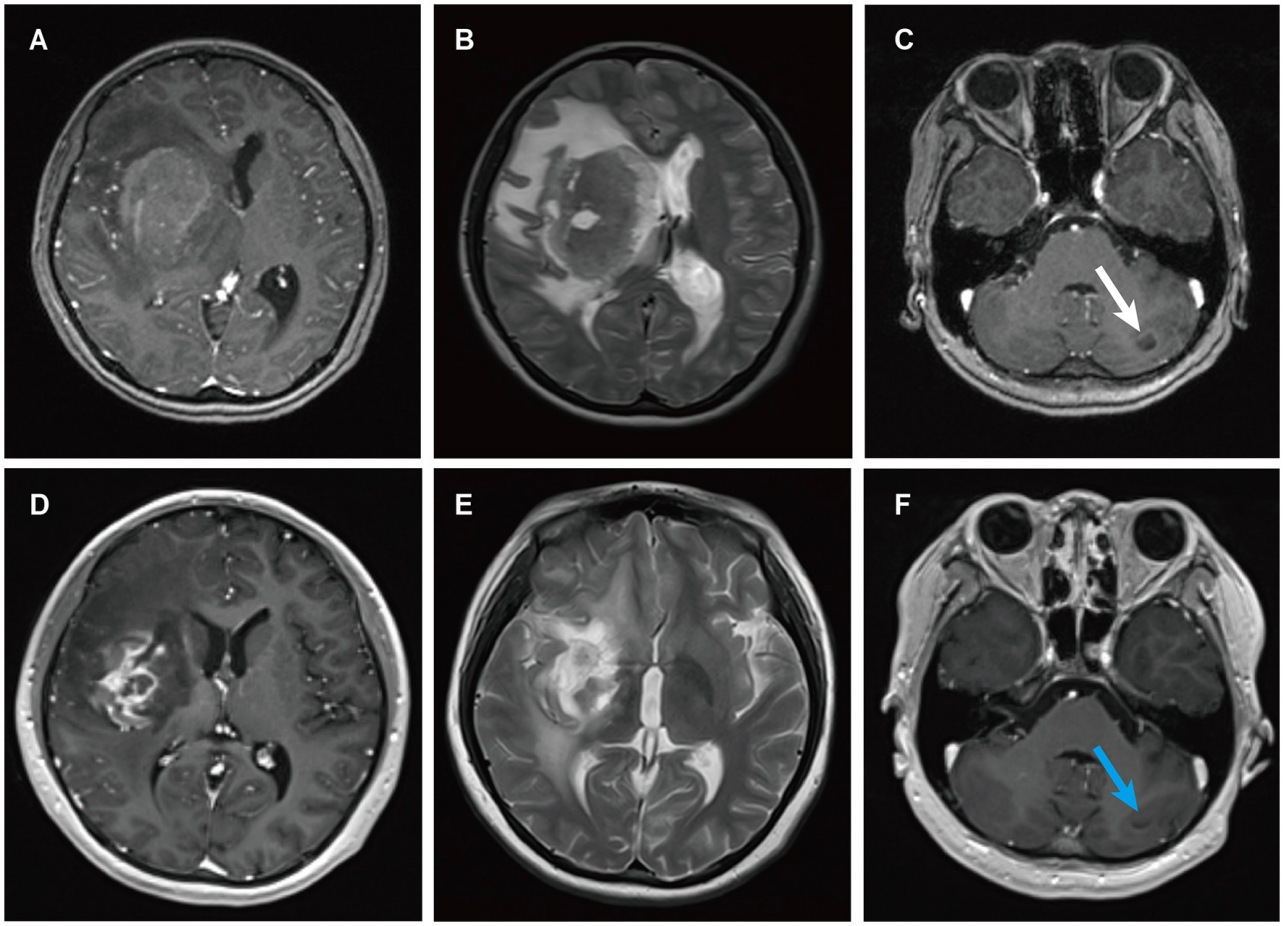
MRI findings of patient 2. Preoperative enhanced MRI prior to brain biopsy showed a ring-enhancing lesion in the right basal ganglia area on T1-weighted images **(A)**, with extensive edema surrounding the lesion in the right basal ganglia area on T2-weighted images **(B)**, and abnormal signal intensity in the left cerebellum on T1-weighted images [**(C)** white arrows]. Enhanced MRI scans obtained four months after the diagnosis of PCNS PTLD, persistent ring enhancement lesion in the right basal ganglia on T1-weighted images **(D)**. Continued perilesional edema in the right basal ganglia on T2-weighted images **(E)**. Additionally, the abnormal signal intensity in the left cerebellum persisted on T1-weighted images [**(F)** blue arrows].

A 13-year-old female presented to a local hospital with a one-month history of facial asymmetry without apparent cause. She underwent allogeneic HSCT for aplastic anemia nine months prior and has been on a regimen of tacrolimus, mycophenolate mofetil, and prednisone as immunosuppressive therapy since then. There is no personal or family history of malignancies. She complained of headaches and left-sided weakness 14 days ago, along with a decrease in mental status and a weight loss of 15 kg over the past nine months. Contrast-enhanced MRI of the head revealed ring-enhancing lesions in the right basal ganglia region and a mass in the left cerebellum (Figure 3). A robot-assisted stereotactic brain biopsy of the right basal ganglia lesion was performed at our department, and histopathological and immunohistochemical studies confirmed the diagnosis of PTLD, consistent with monomorphic diffuse large B-cell lymphoma (DLBCL). Tumor cells stained positive for CD20 and CD79a. EBER *in situ* hybridization was partially positive. CT scans of the neck, chest, abdomen, and pelvis showed no abnormalities. The patient was ultimately diagnosed with PCNS-PTLD. Subsequently, she discontinued all immunosuppressive agents and underwent rituximab therapy. Follow-up MRI of the head four months later showed no significant reduction in the lesion size (Figure 3). Therefore, five months after diagnosis, she underwent tumor resection in the right basal ganglia region at our department. Postoperatively, there was slight improvement in the symptoms of left-sided weakness. Histopathological and immunohistochemical analysis of the brain lesion affirmed the diagnosis of PTLD, exhibiting features characteristic of monomorphic DLBCL, in agreement with earlier brain biopsy results. Tumor cells stained positive for CD20 and CD79a. EBER *in situ* hybridization was positive. She received rituximab and whole-brain radiation therapy (WBRT) after discontinuing all immunosuppressive agents. On the most recent follow-up, there was no recurrence of the disease, and she remained clinically stable.

### 3.3 Case 3

**Figure 4:**
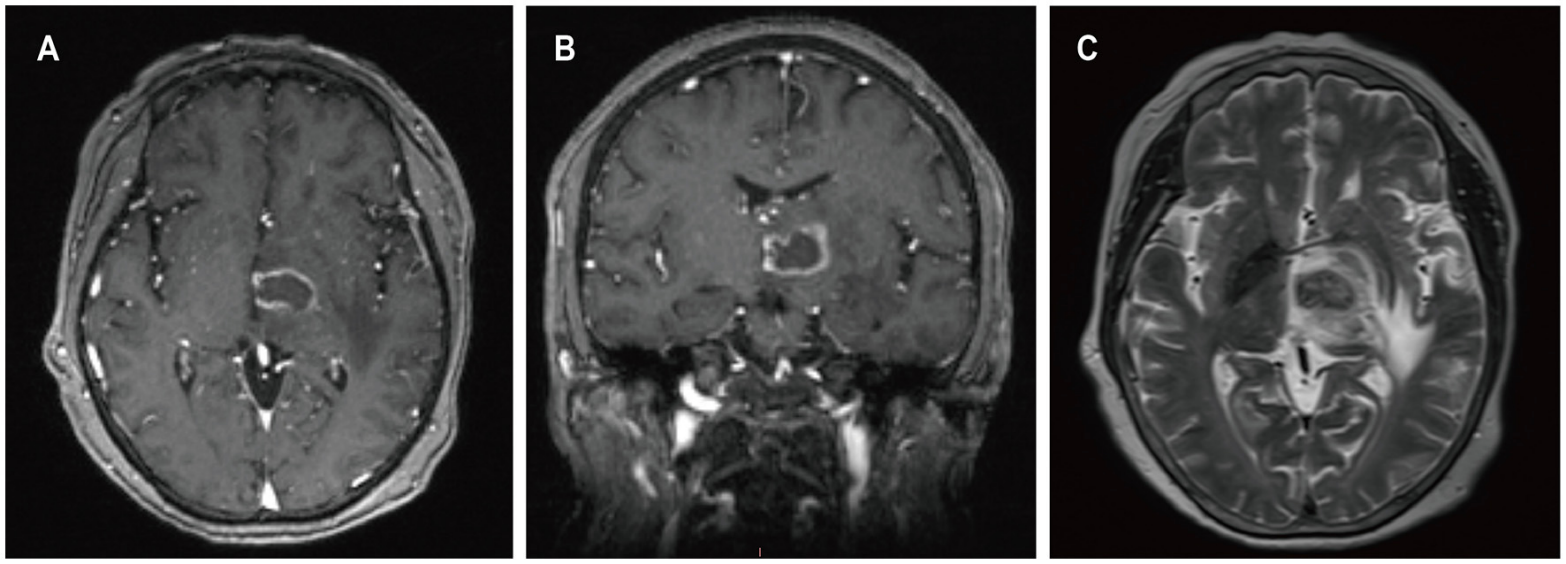
MRI findings of patient 3. Preoperative enhanced MRI prior to brain biopsy showed ring-enhancing lesions affecting the left cerebral peduncle, thalamus, and basal ganglia on T1-weighted images **(A, B)**, with surrounding edema around the left cerebral lesion on T2-weighted images **(C)**.

A 59-year-old female presented with a three-month history of progressive weakness in the right lower limb, characterized by difficulty putting on shoes and walking due to weakness and heaviness in the right leg. She attributed these symptoms to lumbar disc herniation and did not seek medical attention. Two months ago, she began experiencing increased sleep (more than ten hours per day), along with declining memory and forgetfulness, primarily involving recent events. One month ago, she developed weakness in the right upper limb, manifesting as difficulty holding chopsticks and writing. The weakness in the right lower limb worsened, and weakness in the left lower limb developed, while strength in the left upper limb remained normal. She also experienced disorganized speech and naming difficulties. She reported recent low mood and a negative attitude toward things, prompting her to seek treatment at our neurology department. Contrast-enhanced MRI of the head revealed a solitary ring-enhancing lesion involving the left midbrain, cerebral peduncle, thalamus, and basal ganglia (Figure 4). Twenty-four years ago, she underwent kidney transplantation for uremia and has been on a regimen of tacrolimus, mycophenolate mofetil, and prednisone as immunosuppressive therapy since then, with no personal or family history of malignancies. She denied experiencing any symptoms of fever, weight loss, or prodromal signs recently. The neurology department initiated thrombolytic therapy, but there was no improvement in symptoms. Subsequently, she underwent robot-assisted stereotactic brain biopsy of the left thalamic lesion at our neurosurgery department. The pathology report confirmed the diagnosis of PTLD, consistent with monomorphic DLBCL (Hans model indicating non-germinal center origin) (Figure 2). Tumor cells stained positive for CD20 and CD79a. EBER *in situ* hybridization was positive. No significant abnormalities were observed on the CT scans of other body parts. The patient was ultimately diagnosed with PCNS-PTLD. She did not undergo reduction of immunosuppressive agents. Despite treatment with rituximab monotherapy, her condition continued to deteriorate. Approximately three months after being diagnosed with PCNS-PTLD, she passed away due to cardiovascular complications.

### 3.4 Case 4

**Figure 5:**
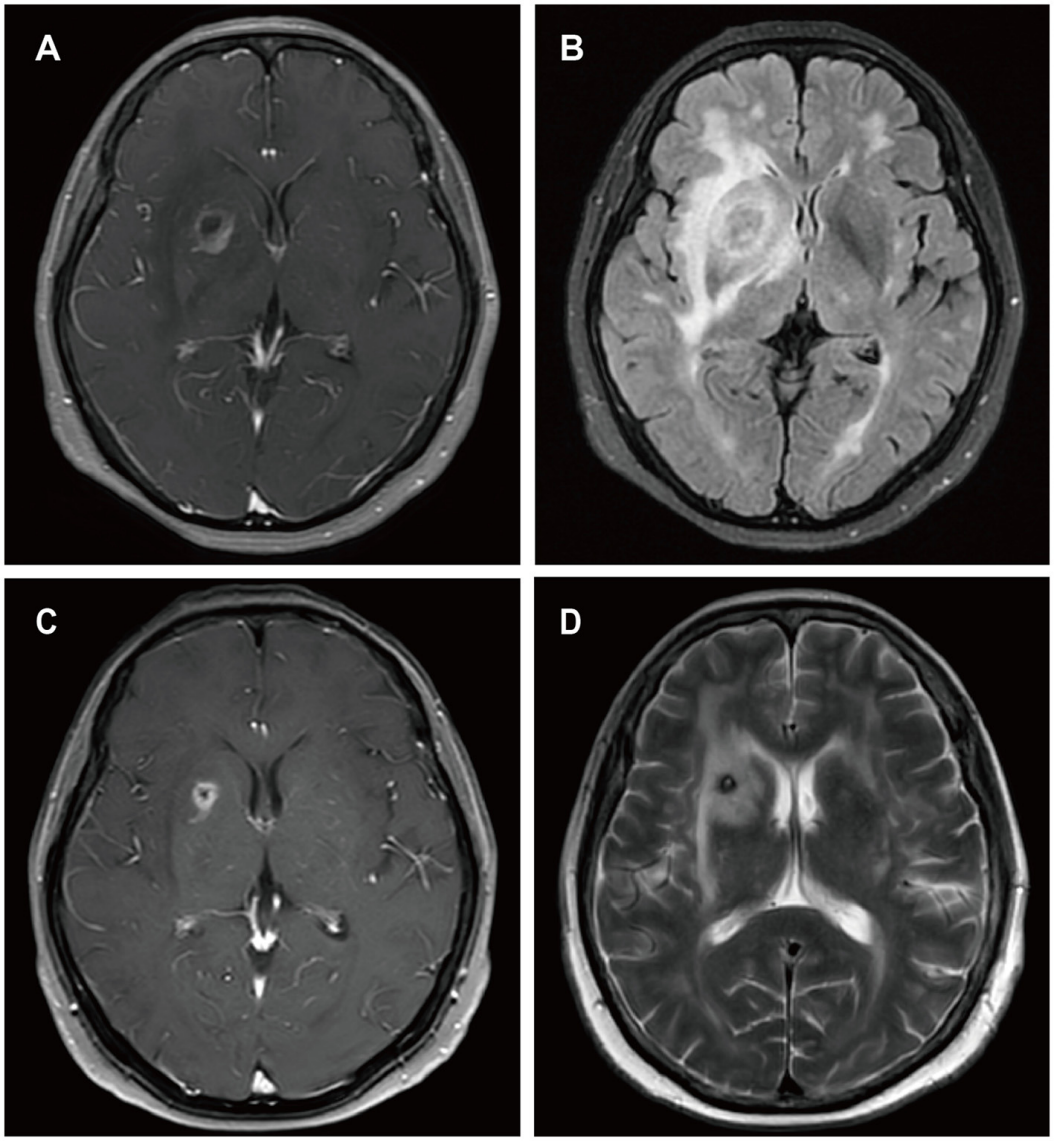
MRI findings of patient 4. Preoperative enhanced MRI prior to brain biopsy showed a ring-enhancing lesion in the right basal ganglia area on T1-weighted images **(A)**, with extensive edema surrounding the lesion in the right basal ganglia area on T2-FLAIR images **(B)**. On follow-up enhanced MRI images obtained 3 months after the diagnosis of PCNS PTLD, the ring-enhancing lesion in the right basal ganglia area appeared reduced on T1-weighted images **(C)**, and the edema surrounding the lesion in the right basal ganglia area decreased on T2-weighted images **(D)**.

A 62-year-old female presented to our hospital with occipital pain persisting for three months, occasional mild headaches, and intermittent nausea. Nineteen years ago, she received a kidney transplant for uremia and has been taking mycophenolate mofetil, cyclosporine, and prednisone ever since, without any personal or family history of malignancies. She denied vomiting, fever, weight loss, weakness in the limbs, or any other prodromal symptoms. A week ago, contrast-enhanced MRI of the head revealed a ring-enhancing lesion in the right basal ganglia (Figure 5). Subsequently, she underwent robot-assisted stereotactic brain biopsy of the right basal ganglia lesion at our department. Histopathological and immunohistochemical studies confirmed the diagnosis of PTLD, consistent with monomorphic DLBCL. Tumor cells stained positive for CD20 and CD79a. EBER *in situ* hybridization was positive. CT scans of the neck, chest, abdomen, and pelvis showed no abnormalities. The patient was ultimately diagnosed with PCNS-PTLD. She underwent reduction of immunosuppressive agents combined with treatment with zanubrutinib. Follow-up contrast-enhanced MRI of the head three months later showed a reduction in the lesion size in the right basal ganglia (Figure 5). On the last follow-up, she remained clinically stable.

### 3.5 Case 5

**Figure 6:**
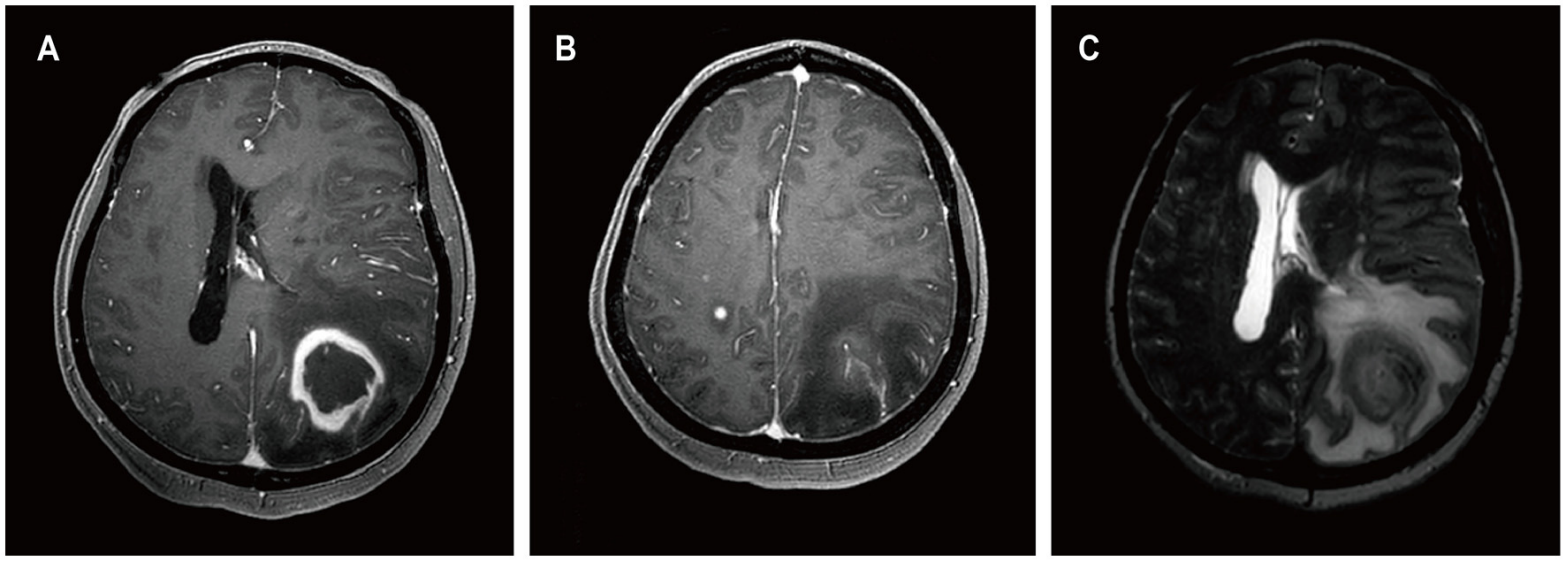
MRI findings of patient 5. Preoperative enhanced MRI images showed a ring-enhancing lesion in the left parietal lobe on T1-weighted images **(A)**, a circular lesion in the right parietal lobe **(B)**, and extensive edema surrounding the lesion in the left parietal lobe on T2-weighted images **(C)**.

A 52-year-old female presented with a 23-day history of cognitive decline characterized by transient inability to comprehend normal behavior and recent memory loss lasting 2-3 minutes, with spontaneous recovery. Seven days prior to admission, she developed weakness in the right lower limb, instability while walking, and a right visual field defect, accompanied by occasional nausea and vomiting, prompting evaluation at a local hospital. Twenty-seven years ago, she underwent kidney transplantation for chronic renal failure and has since been on a regimen of cyclosporine, mycophenolate mofetil, and methylprednisolone as immunosuppressive therapy, with no personal or family history of malignancies. She denied fever, night sweats, numbness in the extremities, and joint pain. Her weight had decreased by 1 kg over the past month. Contrast-enhanced MRI of the head revealed a ring-enhancing lesion in the left parietal lobe with surrounding edema and a circular lesion in the right parietal lobe (Figure 6). Robot-assisted stereotactic brain biopsy of the left parietal lobe lesion was performed at our department. Histopathological and immunohistochemical studies confirmed the diagnosis of PTLD, consistent with monomorphic DLBCL. Tumor cells stained positive for CD20 and CD79a. EBER *in situ* hybridization was positive. PET-CT showed a mixed-density mass in the left parietal lobe with increased glucose metabolism, suggestive of an intracranial primary malignant lesion. The patient was ultimately diagnosed with PCNS-PTLD following kidney transplantation. Mycophenolate mofetil was discontinued, cyclosporine dosage was increased, and methotrexate was initiated. Three days after the diagnosis of PCNS-PTLD, she developed coma due to cerebral edema, and considering the poor prognosis, conservative treatment with mannitol was decided upon in consultation with the patient's family. Serial head CT scans showed worsening cerebral edema, and transcranial Doppler (TCD) ultrasonography indicated severe intracranial hypertension with fixed dilated pupils. She passed away 18 days after the diagnosis of PCNS-PTLD due to cerebral herniation.

## 4 Results

### 4.1 Demographics and transplantation information

In our institution, a total of five patients were diagnosed with PCNS-PTLD (Table 1). Among them, one patient had previously undergone allogeneic HSCT and was identified with PCNS-PTLD at the age of 13, while four patients had undergone kidney transplantation, with a median age of 58 years at the time of PCNS-PTLD diagnosis. In a review of 22 articles (Table 2), 6 articles reported cases of PCNS-PTLD occurring after allogeneic HSCT, while 16 articles documented cases following kidney transplantation. The median age at diagnosis of PCNS-PTLD for patients who had undergone allogeneic HSCT was 40.5 years, whereas for those who had kidney transplants, it was 44 years.

In our case series, patients who had undergone kidney transplantation had a median time of 21.5 years from their last transplant surgery to the diagnosis of PCNS-PTLD, whereas for those who had undergone allogeneic HSCT, this period was only 9 months. This trend is also observed in the 22 reviewed articles, where the median time from the last transplant surgery to PCNS-PTLD diagnosis for patients who had kidney transplants was 7 years, compared to just 8 months for those who had undergone allogeneic HSCT.

Immunosuppressive drugs administered to the patients at diagnosis are shown in Tables 1, 2, respectively.

### 4.2 Presenting symptoms

The clinical symptoms exhibited by the patients were highly variable and included motor weakness (*n* = 4), headache (*n* = 2), nausea (*n* = 2), confusion (*n* = 2), speech difficulties (*n* = 1), depressive mood (*n* = 1), facial droop (*n* = 1), and visual field defect (*n* = 1), as shown in Table 1. These symptoms are consistent with those reported in previous literature, which also mentions additional symptoms such as seizures, diplopia, vertigo, ataxia, and palsy (Table 2).

### 4.3 Imaging findings

All our patients underwent imaging studies preoperatively. Through these imaging examinations, we found that all patients exhibited lesions with ring enhancement (n = 5), with 3 patients presenting with a solitary lesion and 2 with multiple lesions. Specifically, 2 patients had lesions involving both supratentorial and infratentorial regions, while 3 patients had lesions confined to the supratentorial region only, as shown in Table 1. In the review of 22 articles (Table 2), there were 10 patients with solitary lesions and 24 patients with multiple lesions. Among these 22 retrospective articles, 16 mentioned ring-enhancing lesions. Additionally, 24 patients had lesions confined to the supratentorial region, 8 patients had lesions involving both supratentorial and infratentorial regions, and 1 patient had lesions confined to the infratentorial region.

### 4.4 Brain biopsy findings

The diagnosis of PCNS-PTLD in our patients was confirmed through pathological examination of brain tissue obtained via robot-assisted stereotactic brain biopsies (*n* = 4) and surgical resection (*n* = 1). Among these patients, one was diagnosed with polymorphic PTLD, while the remaining four were diagnosed with monomorphic DLBCL (Table 1). In the patients reported in the 22 reviewed articles, 28 were explicitly identified as monomorphic DLBCL, 6 as polymorphic PTLD, with the remaining pathology types detailed in Table 2.

All of our patients exhibited EBV and CD20 positivity in their brain tissue.

### 4.5 Treatments and survival outcomes

In our patient group, four patients initially had a reduction of immunosuppression. The treatment protocols for three patients included rituximab, two underwent surgical resection, one was treated with WBRT, one with zanubrutinib, and one with methotrexate, as shown in Table 1. In the 22 reviewed articles, 11 reported the use of WBRT in treatment, 3 reported surgical resection, and 1 reported the use of ibrutinib, with the rest of the treatment protocols detailed in Table 2.

As of the most recent follow-up, the median follow-up duration was 19 months (range: 18 days to 42 months). Three patients have passed away: two due to complications related to cardiovascular issues, and one as a result of cerebral herniation, which was a consequence of the progression of PCNS-PTLD. The specific survival times are detailed in Table 1. The survival outcomes reported in the 22 reviewed articles are presented in Table 2.

Two of our patients are currently alive and in a clinically stable phase. One patient, diagnosed with PCNS-PTLD after allogeneic HSCT, was treated with withdrawal of all immunosuppression, surgical resection, and a combination of rituximab and WBRT. The second patient, who had a history of kidney transplantation, received a reduction in immunosuppression and a combination therapy including zanubrutinib.

## 5 Discussion

PTLD is a rare complication of SOT or HSCT (13). The etiology of PTLD is multifactorial, involving immunosuppressive drug use, Epstein-Barr virus (EBV) infection, and age (26). For HSCT recipients, factors also include T-cell depletion, HLA mismatch, unrelated donor transplantation, and chronic graft-versus-host disease (GVHD) (26, 27). Typically, a competent host can initiate both humoral immunity through antibody production and cell-mediated immunity through cytotoxic T-cell responses. However, in organ transplant patients, the use of immunosuppressive agents leads to T-cell dysfunction, resulting in loss of T-cell control over B-cell proliferation, leading to uncontrolled proliferation of EBV-transformed B cells (2). Among these drugs, mycophenolate mofetil, tacrolimus, cyclosporine, azathioprine, and corticosteroids are the major medications associated with increased incidence of PTLD (28). In kidney transplant recipients, the incidence of PTLD is approximately 1% (13, 15). In allogeneic HSCT recipients, the incidence is about 1–3% (25), while it is 6.2% in lung transplant recipients, 2% in heart transplant recipients, and 1.4% in liver transplant recipients (29). The variation in incidence rates is related to the specific immunosuppressive regimens for different organs (29). Our study found that the time from the last allogeneic HSCT to the diagnosis of PCNS-PTLD in patients was shorter than that in kidney transplant recipients, a finding that is also confirmed in the reviewed literature. This might be related to the specific immunosuppressive regimen, including the dose of each drug used.

EBV plays a crucial role in the progression of PTLD, with 90% of PCNS-PTLD cases associated with EBV (8). Our reported five patients all tested positive for EBV *in situ* hybridization, confirming this association. During primary infection, EBV immortalizes B lymphocytes, leading to polyclonal activation and proliferation, which is finely balanced by EBV-specific immune control to maintain EBV latency status. However, in immunocompromised hosts, defects in EBV inhibition and cytotoxic function lead to disruption of immune balance, resulting in proliferation of EBV-infected B lymphocytes and ultimately the development of PTLD (30). Currently, there is an increasing trend in the number of EBV-negative PTLD patients, possibly due to new immunosuppressive regimens and increased awareness of EBV-positive risk factors (31). The risk of PTLD is higher in SOT recipients under 10 years old and over 60 years old (26). The incidence of PTLD in children is four times higher than in adults, primarily because children have a higher rate of EBV seronegativity, making EBV-negative children more susceptible to infection from transplanted organs, thereby increasing the likelihood of PTLD development (29). In contrast, the elderly are mainly at risk due to declining immune surveillance capabilities (26).

Diagnosing PCNS-PTLD is often challenging and requires comprehensive physical examination, diagnostic imaging, and histopathological biopsy (2). For EBV-positive PTLD, quantitative polymerase chain reaction (qPCR) testing of EBV DNA is a sensitive early diagnostic tool. Studies suggest that relying on plasma EBV viral load for PTLD diagnosis has a sensitivity of up to 92% (32). Additionally, EBV positivity in cerebrospinal fluid (CSF) also strongly suggests CNS PTLD diagnosis (33). Case reports indicate that in patients with CNS PTLD, CSF EBV can be positive even when blood EBV is negative (34). While blood and CSF EBV DNA testing can support the diagnosis of PCNS-PTLD, final confirmation still relies on histopathological biopsy. Furthermore, there are reports of patients diagnosed with PCNS-PTLD whose EBV is negative in both CSF and blood, highlighting the limitation of relying solely on EBV testing to exclude the disease (35).

Clinical symptoms in PCNS-PTLD patients are highly atypical, usually associated with intracranial lesions, presenting mainly as seizures, neuropsychiatric symptoms, focal neurological deficits, and symptoms of increased intracranial pressure (29). Specifically, patients may experience headaches, nausea, vomiting, limb weakness, hemiparesis, ataxia, gait instability, speech difficulties, confusion, seizures, among other symptoms. Less commonly, facial nerve paralysis can occur (15). Therefore, relying solely on symptomatology for diagnosis or exclusion of PCNS-PTLD is extremely challenging.

Imaging plays a crucial role in the diagnosis of PCNS-PTLD. MRI offers advantages in sensitivity and tissue contrast, making it the preferred imaging modality for evaluating transplant recipients (4). According to the literature review, the imaging characteristics of most PCNS-PTLD lesions are multifocal supratentorial ring-enhancing lesions, although our case series suggests that solitary supratentorial ring-enhancing lesions should also raise suspicion for this disease. Tumors with high cell density are prone to bleeding, cystic changes, necrosis, and surrounding edema (33). Ring enhancement may indicate necrotic lesions in the CNS (25). A study reviewing 221 MRI cases with ring enhancement in CNS found that 40% were gliomas, 30% were brain metastases, 12% were brain abscesses, 6% were multiple sclerosis, and only 2% were lymphomas (36). EBV viral encephalitis can also present as ring-enhancing masses (37). Cases have reported that patients with EBV CNS infection progressed to PCNS-PTLD after 5 months (8). CT scans may show various changes, including high, moderate, and low-density alterations (33). Because PCNS-PTLD patients do not exhibit specific enhancements on CT and MRI scans (17), the presence of supratentorial ring enhancement on MRI, while highly suggestive of PCNS-PTLD, does not exclude other diseases. Definitive diagnosis relies on histopathological biopsy. Recent case reports have indicated that PCNS-PTLD lesions may exhibit high perfusion and elevated levels of choline and lipids, aiding in differentiation from inflammation (16).

Therefore, whether it's the results of plasma and CSF EBV DNA, the clinical presentations of patients, or even imaging findings, they can only assist in the diagnosis of PCNS-PTLD. The final diagnosis requires histopathological biopsy (33). In our cases, 80% of the patients underwent robot-assisted stereotactic brain biopsies based on the ROSA robotic system (Zimmer Biomet Robotics, Montpellier, France) or the REMEBOT domestic neurosurgical robot (Beijing Baihui Weikang Technology Co., Ltd., Beijing, China).

Stereotactic brain biopsy is a minimally invasive technique aimed at obtaining reliable histological diagnoses (38). Traditionally, there are frameless and frame-based methods, each with its own advantages and disadvantages (38). However, stereotactic brain biopsies guided by the ROSA system integrate the strengths of both methods in terms of technique, time efficiency, and diagnostic accuracy (38). The use of robotic systems in neurosurgery has expanded widely following the advent of MRI-guided stereotaxy (39). Improvements in accuracy, safety, and user-friendly modalities such as frameless surface registration and the ROSA system have facilitated the incorporation of robotics into the biopsy process (40). The ROSA system, an image-guided device with advanced navigation and haptic capabilities, allows neurosurgeons to choose between supervising the robot performing autonomously or directly controlling and moving the surgical instruments during the procedure after offline planning (38). The ROSA system enhances the safety and feasibility of stereotactic brain biopsies, while minimizing surgical risks and time (38, 41). Both ROSA and REMEBOT are active arm robotic systems equipped with six degrees of motion freedom. However, compared to ROSA, the REMEBOT system requires less registration time for the procedures it guides (42). Preoperatively, we utilized thin-slice contrast-enhanced MRI and CT for lesion localization in patients. Patients who were unable to undergo MRI due to various contraindications underwent preoperative localization using contrast-enhanced CT. For surgeries requiring prone or lateral positioning, scalp markers were applied to the patient's head prior to preoperative CT examination. Subsequently, the preoperative MRI and CT imaging data were imported into the operation planning subsystem of robot-assisted stereotactic biopsy systems to set the cranial entry point, puncture tract, and biopsy target. Intraoperatively, facial laser scanning or scalp markers were used for registration. Following this, guided by the pre-set biopsy trajectory, biopsies were performed with negative-pressure aspiration under the assistance of the robot's mechanical arm. The biopsy trajectory should steer clear of significant vessels visible on imaging, extract lesion tissue along its longitudinal axis, and avoid piercing into brain ventricles, among other considerations. Care was taken regarding the magnitude of negative pressure during tissue aspiration. Postoperatively, a cranial CT was conducted to confirm the accuracy of the puncture site and to check for complications such as intracranial hemorrhage. Specimens obtained during surgery were sent for pathological examination, and subsequent treatment measures were determined based on the pathological results.

In 2017, the World Health Organization (WHO) categorized PTLD into six subtypes, with three being non-destructive PTLD, including plasmacytic hyperplasia, infectious mononucleosis-like PTLD, and florid follicular hyperplasia (27). The other three are destructive PTLD, including polymorphic PTLD, monomorphic PTLD, and classic Hodgkin lymphoma-like PTLD (27). Combining our cases with other case reports, monomorphic DLBCL appears to be the most common pathological type of PCNS-PTLD.

Due to the limited number of PCNS-PTLD cases and the lack of systematic studies, there is currently no standardized treatment regimen (43). Existing treatment methods include reducing immunosuppressive drug doses, chemotherapy, rituximab therapy, EBV-specific cytotoxic T lymphocyte therapy, surgical resection, and WBRT. Reducing the dose of immunosuppressive drugs is the primary and initial method for treating PTLD (26). However, reducing immunosuppressive drugs alone is often insufficient (43). A retrospective study showed that its effectiveness rate was only 45% (44).

Previous studies have demonstrated that high-dose methotrexate-based chemotherapy regimens in PCNS-PTLD patients post-SOT are not only effective but also well-tolerated (45). High-dose methotrexate is defined as a dose exceeding 500 mg/m^2^ (46). Since 90% of methotrexate relies on renal excretion (46), end-stage renal disease is a contraindication for its use (12).

Although chemotherapy has a high success rate, it carries treatment-related toxicity and mortality rates (43). Additionally, the most common pathological type of PCNS-PTLD is DLBCL derived from B lymphocytes, with CD20 positivity in atypical cells. Rituximab, a monoclonal antibody against CD20 on mature B lymphocytes, induces apoptosis and complement-mediated cytotoxicity against CD20-positive cells (19). Therefore, rituximab is increasingly used (43). Currently, rituximab combined with or without chemotherapy is gradually becoming a first-line treatment option (31).

Recent reports suggest that enhanced high-flux hemodialysis can effectively clear methotrexate from the bodies of end-stage renal disease patients (12, 46). Therefore, in PCNS-PTLD patients with concurrent end-stage renal disease, high-dose methotrexate combined with enhanced high-flux hemodialysis, methotrexate concentration monitoring, and ready-to-use calcium folinate rescue therapy can be attempted (46). However, due to limited related reports, the safety of this method requires further research confirmation.

Furthermore, in our case series, one patient received reduced immunosuppression combined with zanubrutinib treatment, and follow-up MRI revealed a gradual reduction in the tumor mass in the right basal ganglia region (Figure 5). The patient remained clinically stable until the last follow-up visit. Our case suggest that zanubrutinib may also be a potentially safe and effective drug for treating PCNS-PTLD.

Case reports have shown successful treatment of PCNS-PTLD patients using the zanubrutinib induction/maintenance therapy followed by consolidation therapy with third-party specific EBV T lymphocytes for a duration of one year (11). Zanubrutinib is a second-generation Bruton's Tyrosine Kinase inhibitor (BTKi) with superior inhibitory activity, higher bioavailability, and the ability to achieve sustained therapeutic exposure compared to the first-generation BTKi ibrutinib. Moreover, zanubrutinib exhibits significantly improved drug-drug interaction profiles, indicating its potential for concurrent use with a wider range of medications (47). Bruton's Tyrosine Kinase (BTK) plays a crucial role in the intracellular signaling pathway of B lymphocyte receptors, mediating the development, proliferation, and survival of B lymphocytes (47). Aberrant BTK signaling is pivotal in the pathogenesis and progression of various B cell malignancies, including DLBCL (47). Recent studies have demonstrated that treatment regimens incorporating BTK inhibitors, such as zanubrutinib, can safely and effectively treat both systemic high-risk DLBCL and primary central nervous system lymphoma (PCNSL) (48). Given the safety and efficacy of zanubrutinib in the treatment of various B cell malignancies, it has been approved for use in more than 60 countries and regions (47).

Surgical resection remains controversial in PCNS-PTLD treatment. Traditionally, surgery has been discouraged due to the potential occult spread of lymphoma throughout the brain (39). However, emerging evidence suggests that maximal safe resection may be beneficial for immediate relief of tumor mass effects (49). Furthermore, studies have indicated that patients undergoing subtotal or total resection have significantly improved progression-free survival and overall survival compared to those undergoing biopsy alone (50). In our case series, two patients who underwent surgical resection showed no tumor recurrence on postoperative MRI, with one patient showing no signs of recurrence to date and another succumbing to myocardial infarction three years after surgery rather than PTLD. Therefore, our cases tend to support surgical resection.

Known lymphomas or PTLDs exhibit sensitivity to moderate doses (3,000–4,500 cGy) of radiation (4). Additionally, WBRT offers a high complete remission rate, lacks systemic toxicity, and poses no risk of allograft dysfunction (4). Based on our literature review, it can be observed that WBRT is also a commonly employed method for treating PCNS-PTLD.

Case series have shown that WBRT following complete resection can prolong survival in PCNS-PTLD patients (9). Additionally, case report has confirmed that WBRT in combination with rituximab is an effective treatment for PCNS-PTLD (6). In our case series, there was a patient who developed PCNS-PTLD following allogeneic HSCT. She underwent a combination of the above treatments, which confirmed the safety and efficacy of this combined therapeutic regimen.

The prognosis of PCNS-PTLD is poor and depends primarily on the patient's age, severity of the disease at diagnosis, potential complications, risk of allograft dysfunction, and treatment strategies (4, 23). Studies have shown that lack of response to first-line treatment and elevated lactate dehydrogenase levels are associated with a poor prognosis for PCNS-PTLD (1). Timely diagnosis of this disease is crucial for initiating potentially side-effect-ridden long-term targeted therapies (24). Early brain biopsy is necessary for patients highly suspected of having PCNS-PTLD.

Our study has some limitations. Due to a lack of understanding of the disease at the time, we did not perform EBV tests in blood or CSF preoperatively. Additionally, the number of cases we reported, as well as the quantity of relevant literature, is very limited. Therefore, the potential effectiveness of the treatment protocols we identified requires further validation in future studies.

## 6 Conclusion

Diagnosing PCNS-PTLD is extremely challenging and can only be confirmed through histopathological biopsy. Clinical presentations in patients are generally associated with intracranial lesions and are not typical. MRI scans in patients may also show various abnormal signals. However, we have found that supratentorial ring-enhancing lesions are the most common feature of this disease. Therefore, for patients who have undergone kidney or allogeneic HSCT and have been on long-term immunosuppressive therapy, any neurological symptoms and MRI evidence of intracranial abnormal signals, especially supratentorial ring-enhancing masses, whether single or multiple lesions, should raise a high suspicion of PCNS-PTLD. Timely brain biopsy should be performed to help choose specific treatments related to this disease as early as possible. The prognosis of PCNS-PTLD is poor, and due to its rarity, there is currently no recommended standard treatment protocol. Here, we report four cases of PCNS-PTLD after kidney transplantation and one case after allogeneic HSCT, contributing to the limited literature available. Based on our case series, apart from reducing the dosage of immunosuppressants, zanubrutinib may be a safe and effective treatment for this disease. A combined treatment approach with rituximab and WBRT after complete tumor resection is also a potential safe and effective strategy.

## Data availability statement

The original contributions presented in the study are included in the article/supplementary material, further inquiries can be directed to the corresponding authors.

## Ethics statement

The studies involving humans were approved by Xuanwu Hospital Ethics Committee. The studies were conducted in accordance with the local legislation and institutional requirements. Written informed consent for participation in this study was provided by the participants' legal guardians/next of kin. Written informed consent was obtained from the individual(s), and minor(s)' legal guardian/next of kin, for the publication of any potentially identifiable images or data included in this article.

## Author contributions

LJ: Data curation, Formal analysis, Investigation, Software, Writing – original draft. DL: Data curation, Formal analysis, Investigation, Methodology, Writing – original draft. FY: Methodology, Resources, Writing – review & editing. JH: Data curation, Investigation, Writing – review & editing. PW: Conceptualization, Writing – review & editing. YZ: Writing – review & editing, Data curation, Investigation. YW: Funding acquisition, Resources, Supervision, Writing – review & editing, Conceptualization, Methodology. YS: Resources, Supervision, Writing – review & editing, Funding acquisition. GZ: Project administration, Resources, Supervision, Writing – review & editing.
